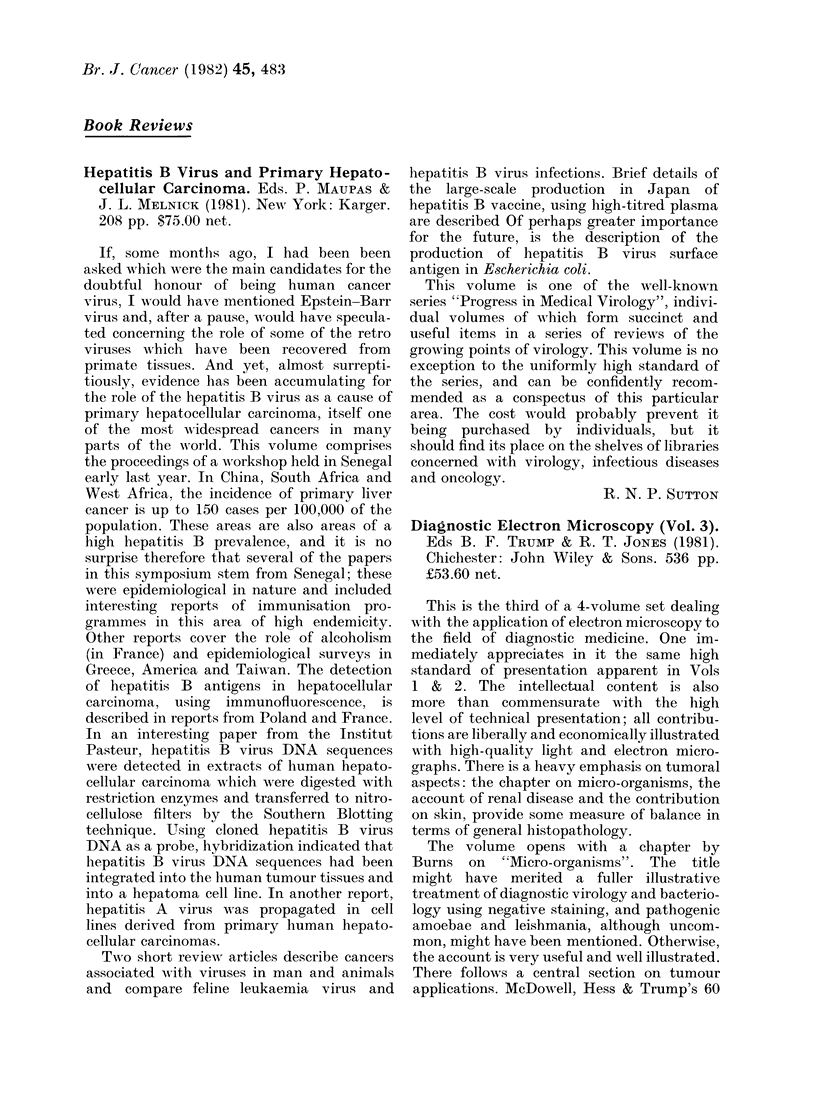# Hepatitis B Virus and Primary Hepatocellular Carcinoma

**Published:** 1982-03

**Authors:** R. N. P. Sutton


*Br. J. Cancer* (1982) **45,** 483

### *Book Reviews*

**Hepatitis B Virus and Primary Hepatocellular Carcinoma.** Eds. P. Maupas & J. L. Melnick (1981). New York: Karger. 208 pp. $75.00 net.

If, some months ago, I had been been asked which were the main candidates for the doubtful honour of being human cancer virus, I would have mentioned Epstein–Barr virus and, after a pause, would have speculated concerning the role of some of the retro viruses which have been recovered from primate tissues. And yet, almost surreptitiously, evidence has been accumulating for the role of the hepatitis B virus as a cause of primary hepatocellular carcinoma, itself one of the most widespread cancers in many parts of the world. This volume comprises the proceedings of a workshop held in Senegal early last year. In China, South Africa and West Africa, the incidence of primary liver cancer is up to 150 cases per 100,000 of the population. These areas are also areas of a high hepatitis B prevalence, and it is no surprise therefore that several of the papers in this symposium stem from Senegal; these were epidemiological in nature and included interesting reports of immunisation programmes in this area of high endemicity. Other reports cover the role of alcoholism (in France) and epidemiological surveys in Greece, America and Taiwan. The detection of hepatitis B antigens in hepatocellular carcinoma, using immunofluorescence, is described in reports from Poland and France. In an interesting paper from the Institut Pasteur, hepatitis B virus DNA sequences were detected in extracts of human hepatocellular carcinoma which were digested with restriction enzymes and transferred to nitrocellulose filters by the Southern Blotting technique. Using cloned hepatitis B virus DNA as a probe, hybridization indicated that hepatitis B virus DNA sequences had been integrated into the human tumour tissues and into a hepatoma cell line. In another report, hepatitis A virus was propagated in cell lines derived from primary human hepatocellular carcinomas.

Two short review articles describe cancers associated with viruses in man and animals and compare feline leukaemia virus and hepatitis B virus infections. Brief details of the large-scale production in Japan of hepatitis B vaccine, using high-titred plasma are described Of perhaps greater importance for the future, is the description of the production of hepatitis B virus surface antigen in *Escherichia coli*.

This volume is one of the well-known series "Progress in Medical Virology", individual volumes of which form succinct and useful items in a series of reviews of the growing points of virology. This volume is no exception to the uniformly high standard of the series, and can be confidently recommended as a conspectus of this particular area. The cost would probably prevent it being purchased by individuals, but it should find its place on the shelves of libraries concerned with virology, infectious diseases and oncology.

R. N. P. Sutton